# Dental Jurisprudence

**Published:** 1899-11

**Authors:** 


ARTICLE VIII.

### DENTAL JURISPRUDENCE.

---

**Read by Dr. H. A. Croll Palmerston, at Ontario Dental Society Meeting, July, 1897.**

Dental Jurisprudence is an old subject and of the greatest importance, yet one of which but little is known by the average practitioner. The "American System of Dentistry" is, to my knowledge, the only work giving any information on the subject. However, the Board of Directors of the college have, quite recently, placed the subject on the curriculum, and now the student on completing his course has, under the lectures of the Hon. David Mills, imbibed the spirit of the legal difficulties with which he may at some unfortunate time be beset.

Dental jurisprudence may be defined, as the science which teaches every branch of dentistry to the purpose and knowledge of the law. In order then to thoroughly understand the subject, a knowledge must be had, on the one hand, of the law and, on the other, of the professional subjects: Anatomy Physiology, Operative Dentistry, Materia Medica, etc. The jurisprudence of dentistry resembles very much that of medicine, with the exception of a few special points which pertain exclusively to the former profession.

The obtaining of the title of L. D. S., grants the holder permission to practice dentistry in all its branches. This legally interpreted means the care of the teeth when sound, their treatment when diseased, and their substitution when lost through any cause. It includes the extraction, filling, replantation, implantation, and transplantation of the teeth, their regulation, the treatment of the gums, alveolar process, antrum and adjacent bone, both by operative and mechanical means. It also includes prescribing for constitutional treatment when such action is necessary in the treatment of abscesses, trigeminal neuralgia, etc. A person therefore must be qualified according to law if he is to pursue the practice of dentistry or he will be doubly liable for damage; firstly, for practicing without a license; secondly, for lack of necessary skill.

According to law a dentist is responsible for his work. When his assistant does not perform the operation prescribed the dentist is responsible if present, but not so if absent. If the patient requests the act of the dentist, such as the extraction of a sound tooth, he assumes the responsibility, but the dentist is responsible as to the manner in which the operation is performed. If the patient is insane the responsibility of the case rests solely with the dentist. The same applies if the dentist uses new instruments or new drugs with which he is comparatively unacquainted. This is a hard rule, but is for the protection of the public and to prevent experiments being performed upon them. Patent nostrums must have a good reputation to be in general favor. When a dentist operates in a manner contrary to any old established opinion he is liable to censure. A dentist is gravely responsible if he operates when he is intoxicated or when he has not the necessary appliances at hand. These are a few of the responsibilities resting upon dentists.

Should a patient bring into court a case against a dentist it would of necessity be for malpractice. This may be defined as improper management of a case, or such treatment as produces injury, or is illegal. From both a dental and legal point of view it may arise from wilfulness, negligence, or ignorance, and subjects the offender to penalties in any of these categories according as error or criminalty is proved.

A case under the head of wilfulness can be entered only when the dentist has expressed malice and an intent to commit wrong.

Negligence may be divided into three degrees:

1. Slight: Where lack of great care and diligence is shown.

2. Ordinary: Where ordinary skill is wanting.

3. Gross: Where total lack of care is shown.

The state of the patient's health makes a difference in the degree of negligence, the law recognizing that more care should be shown a patient in poor than one in good health. A dentist pursuing obsolete methods is held to be negligent. It is of the utmost importance that his instruments be antiseptically clean. Omission of this is a case of negligence of the most inexcusable type. Negligence is due to want of care, want of habit, loss of morals, and indiffence to business. If the patient by ordinary care could have avoided the negligence of the dentist, he can make no case. But if he can establish that he has not been a contributor to the injury, he may proceed to recover damages.

The standard of skill, or ordinary skill, is that which is the result of the acquaintance with the improvements of the day. The standard of skill is supposed to be greater in cities than in towns and villages, owing to the advantages of libraries, the variety of operations, the opportunities for forming societies and attending conventions.

Should the dentist perform an operation, being incompetent, instead of handing it over to some more capable person, he assumes great responsibility. The more difficult the operation, the greater the liability. Should he be lacking in ordinary skill or guilty of negligence or carlessness he is liable for damages; but if the patient has not strictly carried out the instructions, the dentist's liability is removed. If ordinary skill is shown, he enjoys immunity. It is difficult to show that the dentist does not possess ordinary skill, as he possesses a license, to obtain which he must have demonstrated that he possessed ordinary skill. Suits therefore must be entered for negligence. Specialists in any branch are doubly liable as from their proclaiming themselves as specialists, the law supposes and expects them to possess more than ordinary skill.

Diligence must always be shown. It is the duty of every operator to bring into play all the skill he possesses, but no more than ordinary skill is required of him unless he be a specialist.

A case for malpractice cannot be brought into court if twelve months have elapsed since the injury was first noticed. Should one be brought against a dentist, if he can prove that he has acted honestly in treating the case, and has not thrust himself in the way of a competent person, he is wholly irresponsible. It is necessary that gross negligence or ignorance be shown. The fact that the dentist had no intent to be negligent does not absolve him, as in the eye of the law the deed shows the intent. Where competent aid may be had, a violent remedy given alone involves criminal responsibility. A consultation relieves the dentist of this.

When sued for malpractice the dentist must give the nature of the case, whether acute or chronic; the state of the case when treatment was commenced; his course of treatment; the treatment called for and the opinion of other practitioners. He should prove that there was no negligence, and, if possible that there was delay in seeking aid. If he can prove that he had made a proper diagnosis and given proper treatment, and that all the trouble was due to unforseen constitutional disturbances, the jury will be influenced by a thoroughly well posted counsel.

The consideration in fixing damages are :

1. The extent of the injury.
2. The pain the patient has undergone.
3. The subsequent effect upon the patient's health.
4. The pecuniary loss to the patient.

Where a patient dies in consequence of injuries from negligence or lack of skill, the amount of damages is not to exceed that which the patient could, if living, earn in three years.

Dentists are in some cases summoned as expert witnesses. Under this heading they are allowed extra fees. A dentist is supposed to keep secret the transactions between himself and his patients. In criminal cases the dentist gives the facts of the case in court without breach of honor. The expert witness is allowed to refer to his memoranda, which need not be in his own handwriting, but may not refer to text-books. He tells what he believes, the ordinary witness what he knows. He should avoid technicalities, making everything clear, and should never express an opinion on any subject with which he is not perfectly familiar or he may get slightly mixed.

In Canada a patient is not liable to pay for a piece of work costing over $40.00 if there is no written agreement, part payment or exchange of some article in lieu of cash. A dentist cannot be compelled to render service to a patient when requested, but when once he has taken charge of a case he must continue his services until the case is completed or he is dismissed. A dentist has a right to charge for time lost by an unfilled appointment. The circumstances of the case and the evidence will influence the decision. He also has the right to retain a set of teeth made by him as security for reasonable charges. This right is waived by parting with them or agreeing to give credit for them. Legally no limit is placed upon dentists' fees. They regulate themselves according to the reputation of the operator, the difficulties of the case, and the circumstances in general.

I have above endeavored to give a *resume* of the law governing us in the practice of our profession. Of necessity much was omitted, but the whole of the subect requires a paper which would necessitate an entire day's reading. I trust that I have presented the subject in a manner that some one may have been able to grasp a few heretofore unknown facts.

